# Development of a Quality Assessment Index System for Palliative Care Services in Chinese Nursing Homes: A Modified Delphi and Analytic Hierarchy Process Study

**DOI:** 10.1155/jonm/6031056

**Published:** 2026-07-06

**Authors:** Lu Zhang, Xiao-Qin Wu, Ling Tan, Ping-Pin Wen, Zhi-Xiang Sun, Mei-Fang Yang, Yue-Lin Huang, Jing Fu

**Affiliations:** ^1^ School of Nursing, Southwest Medical University, Luzhou, Sichuan, China, scu.edu.cn; ^2^ School of Clinical Medicine, Southwest Medical University, Luzhou, Sichuan, China, scu.edu.cn; ^3^ Orthopaedic Centre, Second People’s Hospital of Yibin, Yibin, Sichuan, China, scu.edu.cn; ^4^ Department of Otolaryngology-Head and Neck Surgery, The Affiliated Hospital of Southwest Medical University, Luzhou, Sichuan, China, scu.edu.cn; ^5^ Department of Geriatrics, The Affiliated Hospital of Southwest Medical University, Luzhou, Sichuan, China, scu.edu.cn; ^6^ Department of Nursing, The Affiliated Hospital of Southwest Medical University, Luzhou, Sichuan, China, scu.edu.cn

**Keywords:** analytic hierarchy process, Delphi technique, nursing homes, palliative care, quality indicators, structure–process–outcome model

## Abstract

**Background:**

As the population ages, nursing homes are key end‐of‐life care settings, making high‐quality palliative care essential for safeguarding older adults’ quality of life. However, China currently lacks a unified and systematic tool for evaluating the quality of palliative care services in nursing homes.

**Aim:**

To develop a quality assessment index system for palliative care services tailored to Chinese nursing homes.

**Methods:**

The study employed a mixed‐methods design with three phases: (1) a literature review (40 studies, 36 policy/guideline documents; 2013–2023) and semistructured interviews with stakeholders from 6 nursing homes; (2) three rounds of modified Delphi expert consultation (*n* = 31, 27, 23) to revise and refine the indicators; and (3) application of the analytic hierarchy process (AHP) to determine indicator weights.

**Results:**

The final index system comprises 3 first‐level, 22 second‐level, and 47 third‐level indicators. The expert authority coefficient (Cr) exceeded 0.85 across all consultation rounds. Expert consensus increased with each round (Kendall’s W: 0.189 to 0.315, p < 0.05). AHP results indicated that among the first‐level indicators, “process” had the highest weight (0.4114), followed by “structure” (0.3208) and “outcome” (0.2678). Within these dimensions, the highest weighted second‐level indicators were “human resources planning and management” (0.0836), “comfort care services” (0.0861), and “quality of symptom management” (0.0438). Furthermore, key third‐level indicators included “financial support and management” (0.0632), “basic nursing services” (0.0465), and “quality of symptom assessment and management” (0.0438).

**Conclusion:**

This study developed a scientifically grounded quality assessment index system for palliative care services in Chinese nursing homes, which offers a preliminary framework for institutional self‐evaluation and quality improvement efforts pending further empirical validation.

**Implications for Nursing Management:**

Once empirically validated, the framework may assist managers and staff in nursing homes to identify service gaps, optimize resource allocation, and support a shift toward an “older adult–centered” holistic care model.

## 1. Introduction

Global population aging represents a defining demographic trend of the 21st century. By the end of 2024, China’s population aged 65 and above reached 220.23 million, constituting 15.6% of the total population [1]. Projections indicate that this proportion will rise to 29.8% by 2050 [2]. This demographic transition presents substantial challenges to healthcare systems, particularly in the provision of palliative care. Concurrently, significant changes in family structure, including an increase in single‐child and childless families, have led to smaller household sizes and a decline in traditional family‐based care. Consequently, a growing number of older adults are opting for institutional care [3]. Currently, China has approximately 406,000 nursing homes that accommodate over 2 million older residents [1]. These facilities, which offer daily assistance and basic medical care, are increasingly serving as primary residences for older residents who are incapacitated, partially incapacitated, or terminally ill, as they approach the final stage of life [4].

In this context, systematically building palliative care capacity in nursing homes is essential to preserve the quality of life for older residents during the terminal phase [5, 6]. Palliative care aims to alleviate physical and psychological suffering for patients with life‐limiting illnesses while preserving their dignity and quality of life through effective symptom management and tailored support that reflects the needs, values, and cultural backgrounds of patients and their families [7, 8]. However, these institutions face several persistent challenges, with a scarcity of specialized human resources as the primary issue. In many nursing homes, healthcare professionals lack systematic training in palliative care [9]. Participation from multidisciplinary teams, including social workers, psychologists, and rehabilitation therapists, also remains limited [10]. This limitation constrains overall service capacity and professionalism. Consequently, care focuses primarily on physical needs. Individualized psychological, social, and spiritual aspects are neglected [11]. The needs of family members for emotional support, shared decision‐making, and bereavement care are also commonly overlooked [12, 13]. Furthermore, insufficient supportive systems and unclear frameworks present additional barriers. These challenges include limited financial resources, incomplete institutional policies, and ambiguous management protocols [9]. These factors hinder the standardization and advancement of palliative care services.

Establishing a scientific quality assessment index system is essential for promoting service quality improvement. Despite this necessity, research in this area remains at an exploratory stage in China. At the policy level, while China has introduced the Palliative Care Practice Guideline (2025 Edition) [14] and local standards have been developed in certain regions [15], a unified national assessment framework is still lacking. Existing research predominantly addresses quality indicators for palliative care in hospital, community, and home settings [16–18], with limited systematic tools tailored for nursing homes. In contrast, international research has progressed further. For example, Temkin‐Greener et al. [19] developed quality indicators for nursing home palliative care that encompass physiological, psychological, social, and ethical dimensions, as well as multidisciplinary team development and staff training. Thompson et al. [20] introduced the Auditing Care at the End of Life (ACE) tool for nursing homes, which evaluates effectiveness via objective data on symptoms, death preparation, advance care planning, and mortality‐related factors. However, these international approaches are embedded within their respective healthcare systems and cultural contexts, limiting their applicability in China. Consequently, developing a quality assessment framework for palliative care in Chinese nursing homes that aligns with scientific standards and local realities constitutes an urgent research priority.

The creation of a multidimensional evaluation system requires a systematic and rigorous theoretical foundation. Donabedian’s [21] “structure–process–outcome” model provides a classic framework for healthcare quality assessment by delineating three interrelated components. The “structure” dimension encompasses the resources and organizational conditions for care; the “process” dimension includes all activities involved in delivering and receiving care; and the “outcome” dimension refers to the effects on patient and population health. Analyzing these components clarifies the development and quality of services. This model has been extensively validated and applied across various healthcare settings [22–24]. Guided by this framework, the present study employs a mixed‐methods approach to develop a quality assessment index system for palliative care services in Chinese nursing homes. The aim is to provide a scientific basis for standardized evaluation, quality monitoring, and systematic improvement.

## 2. Methods

### 2.1. Research Design

**FIGURE 1:**
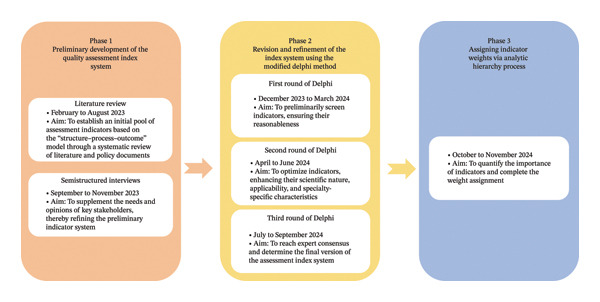
Study flowchart for developing the quality assessment index system.

A mixed‐methods design was employed, utilizing Donabedian’s “structure–process–outcome” model to construct this index system. The research comprised three phases: the development of a preliminary framework through a literature review and semistructured interviews; the refinement and revision of indicators using three rounds of expert consultation via the modified Delphi method; and the determination of indicator weightings through the analytic hierarchy process (AHP). The study received approval from the Ethics Review Committee of Yibin Second People’s Hospital (Approval No. 2023‐220‐01), and all participants provided written informed consent. Figure 1 presents the study flowchart for developing the quality assessment index system.

### 2.2. Research Team

The research team consisted of 11 members, including palliative care researchers from academic institutions and healthcare professionals working in nursing homes and medical facilities. Team members held professional titles, such as Professor, Associate Professor, Lecturer, Senior Nurse, and Teaching Assistant, and had earned doctoral, master’s, or bachelor’s degrees. The team contributed to all stages of the research, including the study design, literature review, indicator extraction, semistructured interviews, questionnaire development, expert opinion analysis, indicator revision, and weight allocation.

### 2.3. Phase One: Preliminary Development of the Quality Assessment Index System

#### 2.3.1. Literature Review

We constructed an initial item pool through a systematic review (protocol preregistered at https://osf.io/wfvek). We systematically searched PubMed, Web of Science, Cochrane Library, CNKI, and Wanfang for studies published between January 2013 and August 2023. The search strategy focused on core concepts, including “nursing home,” “aged,” “palliative care,” “quality indicator,” and their synonyms. Literature screening and data extraction were independently conducted by two researchers. From 7341 initial records, 40 studies were included. Supplementary searches of government and professional society websites yielded 36 relevant policy and guideline documents. Based on Donabedian’s model, we extracted an initial pool comprising 3 first‐level, 11 second‐level, and 40 third‐level indicators with operational definitions. This initial item pool is detailed in Supporting Information 1.

#### 2.3.2. Semistructured Interviews

Between September and November 2023, we conducted semistructured interviews using purposive sampling at six nursing homes in Luzhou and Yibin that provided palliative care. Respondents included facility administrators, professional caregivers (medical staff and nursing aides), residents, and family members. Separate interview guides were developed for each group, covering service content, challenges encountered, service deficiencies, quality concerns, and suggestions for quality assessment indicators. The full interview guides are provided in Supporting Information 2. Sampling continued until data saturation was reached. Each interview lasted approximately 30 min and was audio‐recorded. The transcripts were independently analyzed by two researchers via the seven‐step analysis method of Colaizzi [25]. Seven preliminary quality assessment indicators were identified: financial support, staff qualifications and training management, satisfaction, symptom management, adverse events, palliative care education for families and residents, and refinement of palliative care service systems. Respondent demographics and interview findings are provided in Supporting Information 3. These indicators were integrated with those from the literature review, thereby establishing the initial version of the quality assessment index system.

### 2.4. Phase Two: Revision and Refinement of the Index System Using the Modified Delphi Method

The Delphi method is a well‐established and validated structured group communication process [26]. This approach gathers expert opinions through a series of questionnaires in an anonymous, iterative, and controlled manner, enabling participants to express their views freely after receiving feedback and ultimately achieving expert consensus [22, 27]. The Delphi method is widely employed internationally for developing healthcare quality indicators, with demonstrated reliability in its results [28]. We modified the classical process in two ways: presenting experts with a structured indicator list in the initial round and conducting all consultations online.

#### 2.4.1. Expert Selection

Purposive and snowball sampling methods were employed to identify potential experts from hospitals, nursing homes, government departments, palliative care academic organizations, literature reviews, professional networks, and academic websites. The inclusion criteria were as follows: (1) had five or more years of experience in palliative care/oncology nursing/geriatric care and medicine, nursing management, nursing education, or nursing home management; (2) had a bachelor’s degree or higher; (3) had an intermediate or higher professional title; (4) demonstrated interest in this research and willingness to participate throughout the consultation process; and (5) had a basic understanding of the Delphi consultation method, familiarity with its research methodology, and commitment to sustained participation. Delphi panel sizes typically range from 15 to 50 experts [29]. This study ultimately aimed to include 35 experts. These criteria were selected in line with the flexible and purpose‐oriented nature of expert selection in Delphi studies.

#### 2.4.2. Consultation Process

Three rounds of Delphi expert consultations were conducted via email and WeChat between December 2023 and September 2024. Each round had a 2‐3‐week response period, with reminders sent to nonrespondents. The questionnaire comprised four sections: (1) Introduction: Outlining the study background, content, and objectives while clarifying the anonymous nature of the survey and data confidentiality; (2) Consultation form for the quality assessment index system of palliative care services in nursing homes: The form included columns for indicator names and operational definitions, importance ratings, expert modifications, and an additional comments section for “other opinions or suggestions”; experts assessed the importance of indicators via a 5‐point Likert scale (1 = “not important” to 5 = “very important”) and provided open‐ended feedback on the appropriateness and clarity of the indicators and their operational definitions; (3) Basic expert profile survey: This survey included gender, age, educational background, city of residence, professional title, field of work, and years of experience; and (4) Expert authority assessment: Evaluated experts’ familiarity with the research topic and the basis for judgment. Familiarity is rated on a scale of 1.0 (very familiar) to 0.2 (not at all familiar). Judgment bases are quantified across four dimensions: “high,” “medium,” and “low.” “Practical experience” scores 0.5, 0.4, and 0.3 points; “theoretical analysis” scores 0.3, 0.2, and 0.1 points; and “reference to domestic/international literature” and “expert intuition” score 0.1, 0.1, and 0.1 points.

After questionnaire retrieval, the research team applied screening criteria: mean importance > 3.5 and coefficient of variation (CV) < 0.25 [30]. They then retained the qualifying indicators. The team analyzed expert feedback, revising indicators and operational definitions for subsequent rounds. Ambiguous responses were clarified via consultation. The process ended when substantial consensus was reached. This study adhered to the Standards for Conducting and Reporting Delphi Studies (CREDES) [31].

### 2.5. Phase Three: Assigning Indicator Weights via AHP

The AHP proposed by Saaty constructs a ratio scale through pairwise comparisons and decomposes complex problems into multiple levels, using numerical trade‐offs to assess the relative importance of factors within a hierarchical structure [32]. Consequently, the AHP was employed in this study to determine the weight of each indicator.

#### 2.5.1. Establishing the Hierarchical Model

The AHP divides the overall decision objective into three levels: goal, criteria, and alternatives [32]. In this study, the goal level was defined as “indicators for evaluating the quality of palliative care services in nursing homes.” The criterion level comprised the first‐level and second‐level indicators, whereas the alternative level corresponded to the third‐level indicators.

#### 2.5.2. Constructing the Judgment Matrices

In the third round of expert consultation, an AHP questionnaire was introduced. Using Saaty’s 9‐point rating scale, the questionnaire facilitated the construction of a judgment matrix [33]. The experts assigned scores ranging from 1 to 9 for each indicator pair on the basis of their professional experience, reflecting the relative importance of one indicator compared with another. Specifically, a score of 1 indicates “equally important,” 3 indicates “slightly more important,” 5 denotes “significantly more important,” 7 indicates “much more important,” and 9 signifies “absolutely more important.” Intermediate values of 2, 4, 6, and 8 represent compromise judgments between adjacent scores.

### 2.6. Statistical Methods

Statistical analysis was performed via Excel and SPSS 21.0 software. The demographic characteristics of the experts were presented as frequencies (n) and percentages (%). Expert engagement was represented by the valid questionnaire return rate. Expert authority was quantified via the authority coefficient (Cr), which was calculated as the arithmetic mean of the basis of the judgment score (Ca) and the familiarity score (Cs): Cr = (Ca + Cs)/2. A Cr value ≥ 0.7 was considered acceptable [34]. The concentration of expert opinions was assessed by the mean importance score, standard deviation, and full‐score rate of each indicator. The consistency of expert opinions was evaluated via the CV and Kendall’s coefficient of concordance (W). The W value ranges from 0 to 1, with values closer to 1 indicating greater consistency [35]. A *p* value < 0.05 was considered statistically significant.

The AHP was applied to calculate indicator weights. First, the geometric mean for each row of the judgment matrices was computed. These values were then normalized to obtain the local weights and the global weights. The validity of each judgment matrix was evaluated through a consistency test, which involved calculating the maximum eigenvalue (λmax), the consistency index (CI = (λmax−n)/(n−1)), and the consistency ratio (CR = CI/RI). A CR value < 0.1 was considered acceptable [36]. Otherwise, the matrix was revised iteratively until the CR met this criterion.

## 3. Results

### 3.1. Demographic Characteristics of Experts

Among the 35 experts initially invited, 31 agreed to participate and completed the first‐round Delphi consultation. The panel represents 13 provinces and municipalities across China, with the majority (*n* = 15) from Sichuan Province. Other participants included experts from Guangdong, Hubei, Beijing, and Shanghai (*n* = 2 from each), as well as eight other provinces (one expert from each). The panel consisted of senior clinical specialists (e.g., chief physicians and nurses) from eight hospitals and professors/associate professors from ten universities. All three consultation rounds were completed, with 27 and 23 experts participating in the second and third rounds, respectively. Table 1 summarizes the detailed demographic profile of the experts in each round.

**TABLE 1:** Demographic characteristics of the expert panel in the three‐round modified Delphi study.

Item	Grouping	First round (*N* = 31)		Second round (*N* = 27)		Third round (*N* = 23)	
		Frequency (n)	Proportion (%)	Frequency (n)	Proportion (%)	Frequency (n)	Proportion (%)
Gender	Male	6	19.35	5	18.52	5	21.74
	Female	25	80.65	22	81.48	18	78.26

Age (years)	≤ 40	6	19.35	6	22.22	5	21.74
	41 ∼ 50	11	35.48	9	33.33	7	30.43
	51 ∼ 60	12	38.71	10	37.04	9	39.13
	≥ 61	2	6.45	2	7.41	2	8.70

Highest education	Bachelor’s degree	13	41.94	11	40.74	9	39.13
	Master’s degree	10	32.26	9	33.33	9	39.13
	Doctoral degree	8	25.81	7	25.93	5	21.74

Professional title	Junior	0	0.00	0	0.00	0	0.00
	Intermediate	0	0.00	0	0.00	0	0.00
	Associate senior	12	38.71	9	33.33	7	30.43
	Senior	19	61.29	18	66.67	16	69.57

Field of work (multiple selections permitted)	Nursing management	12	38.71	12	44.44	12	52.17
	Clinical nursing	10	32.26	9	33.33	9	39.13
	Nursing education	12	38.71	10	37.04	10	43.48
	Public administration	3	9.68	2	7.41	2	8.70
	Nursing research	8	25.81	8	29.63	8	34.78
	Clinical medicine	2	6.45	2	7.41	2	8.70
	Medical education	2	6.45	1	3.70	1	4.35

Experience in geriatric/palliative care (years)	5 ∼ 10	5	16.13	4	14.81	4	17.39
	11 ∼ 15	14	45.16	12	44.44	11	47.83
	16 ∼ 20	6	19.35	5	18.52	4	17.39
	21 ∼ 25	2	6.45	2	7.41	1	4.35
	≥ 26	4	12.90	4	14.81	3	13.04

### 3.2. Expert Engagement and Authority

During the three rounds of expert consultation, 35, 31, and 27 questionnaires were distributed, with 31, 27, and 23 valid responses received, respectively. Of the 35 experts initially invited, 4 did not respond to the first‐round questionnaire and were therefore not included in the Delphi panel. Among the 31 experts who participated in the first round, 8 did not complete all subsequent rounds. The reasons included nonresponse despite follow‐up contacts (*n* = 7) and time constraints related to heavy workload (*n* = 1). The corresponding valid response rates were 88.57%, 87.10%, and 85.19%, respectively. All rates exceeded 70%, indicating a high level of expert engagement in this study [37]. The Cr values were 0.877, 0.865, and 0.859 for the three rounds, respectively, all exceeding the acceptable threshold of 0.7, as detailed in Table 2. This indicates a high level of expert authority in this study.

**TABLE 2:** Expert authority in the three‐round Delphi consultation.

Survey round	Ca	Cs	Cr
1	0.913	0.840	0.877
2	0.900	0.830	0.865
3	0.891	0.826	0.859

### 3.3. Concentration and Consistency of Expert Opinions

The results showed that in the first round, the mean importance scores of all the indicators ranged from 3.871 to 4.968, with standard deviations between 0.180 and 1.366, and the full‐score rates ranged from 41.94% to 96.77%. In the second round, the mean scores ranged from 3.667 to 4.963, the standard deviations ranged from 0.193 to 1.375, and the full‐score rates ranged from 37.04% to 96.30%. In the third round, the mean scores ranged from 4.174 to 5.000, the standard deviations ranged from 0.000 to 0.822, and the full‐score rates ranged from 34.78% to 100.00%. Overall, the descriptive statistics suggest a high degree of concentration in expert ratings for most indicators across the three rounds.

The Kendall’s coefficients of concordance (W) for the three rounds were 0.189, 0.266, and 0.315, respectively, which were statistically significant (*p* < 0.05). The significant and progressively increasing W values demonstrate strengthened consensus among experts throughout the iterative consultation process. The consistency of expert opinions across the three Delphi rounds is detailed in Table 3.

**TABLE 3:** Consistency of expert opinions across the three rounds of the Delphi consultation.

Survey round	Number of participants	Indicator	Kendall’s W coefficient	*χ* ^2^	*p* value
First round	31	First‐level indicators	0.302	18.730	< 0.001
		Second‐level indicators	0.214	66.390	< 0.001
		Third‐level indicators	0.168	198.160	< 0.001
		All indicators	0.189	304.808	< 0.001

Second round	27	First‐level indicators	0.233	12.560	0.002
		Second‐level indicators	0.322	190.996	< 0.001
		Third‐level indicators	0.271	380.953	< 0.001
		All indicators	0.266	559.535	< 0.001

Third round	23	First‐level indicators	0.242	11.143	0.004
		Second‐level indicators	0.310	149.650	< 0.001
		Third‐level indicators	0.322	341.006	< 0.001
		All indicators	0.315	513.649	< 0.001

### 3.4. Indicator Screening and Modification

Following the first round of expert consultation, seven third‐level indicators were removed on the basis of expert feedback and predefined screening criteria. Some indicators were redefined, expanded, or merged, leading to the addition of 12 second‐level and 20 third‐level indicators. For example, “B2—respect in service delivery” was expanded into “B4—palliative care education services,” “B5—psychological support and humanistic care,” and “B6—post‐death arrangements and bereavement care services”; the process dimension indicator “B1.5—multidisciplinary team collaboration and communication” was integrated into the operational definition of the structural dimension indicator “A1.1—staffing and workforce management.”

Following the second round of expert consultation, four second‐level indicators that failed to meet the screening criteria and six associated third‐level indicators were removed, and three new second‐level indicators were added. This revision focused on enhancing palliative care specialization, such as refining “B4—palliative care education services” into the more targeted “B4—death education services,” and, on the basis of policy and literature, further specifying the configuration requirements for dedicated spaces under “A2.2—palliative care–related rooms,” including hospice rooms, meeting rooms, and farewell rooms (e.g., usable area, warm design, and facilities).

By the third consultation round, expert opinions had converged, resulting in a stabilized index system. This phase primarily involved refining the nomenclature and operational definitions of indicators without altering their total number. The final quality assessment index system for palliative care services in Chinese nursing homes comprises 3 first‐level, 22 second‐level, and 47 third‐level indicators. These finalized indicators demonstrated mean importance scores ranging from 4.174 to 5.000, CV between 0.000 and 0.191, and full‐score rates ranging from 34.78% to 100.00%. These statistical results reflect a strong consensus among experts, supporting the content validity of the index system. The detailed results of the third‐round consultation and the complete list of finalized indicators are provided in Supporting Information 4 and 5, respectively.

### 3.5. Final Index System With Indicator Weights

The weights for the final index system were determined via the AHP. A total of 19 judgment matrices were constructed, all of which exhibited a CR below 0.1, confirming strong internal consistency. The weight ranges across levels were as follows: first‐level indicators (0.2678–0.4114), second‐level indicators (0.0378–0.0861), and third‐level indicators (0.0051–0.0852), demonstrating good discriminative validity.

**FIGURE 2:**
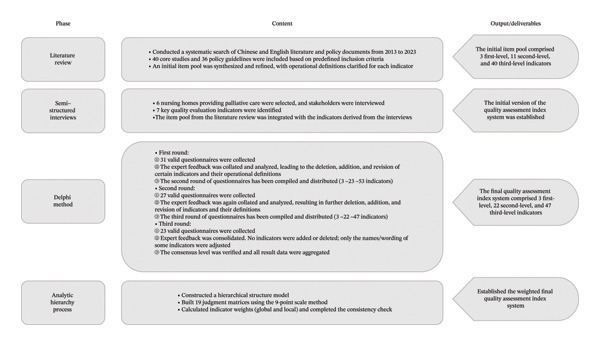
Summary of key processes and outcomes in each research phase.

Weight analysis revealed that among the first‐level indicators, “process indicators” had the highest weight (0.4114), followed by “structural indicators” (0.3208) and “outcome indicators” (0.2678). Within the structural dimension, the second‐level indicator “human resources planning and management” had the highest weight (0.0836), and its subordinate third‐level indicator “staffing and workforce management” carried the greatest weight (0.0316). Within the process dimension, the second‐level indicator “comfort care services” held the highest weight (0.0861), with its third‐level indicator “basic nursing services” being the highest weighted (0.0465). In the outcome dimension, the second‐level indicator “quality of symptom management” had the highest weight (0.0438), and its corresponding third‐level indicator “quality of symptom assessment and management” carried the same weight (0.0438). The complete weight distribution of the index system is detailed in Table 4. Figure 2 summarizes the process and key outcomes of each research phase.

**TABLE 4:** Finalized palliative care quality assessment index system with weight distributions.

First‐level indicators	Global weight	Second‐level indicators	Local weight	Global weight	Third‐level indicators	Local weight	Global weight
A. Structural indicators	0.3208	A1. Human resources planning and management	0.2605	0.0836	A1.1. Staffing and workforce management	0.3785	0.0316
			0.2605	0.0836	A1.2. Personnel qualification requirements	0.3104	0.0259
			0.2605	0.0836	A1.3. Training and management	0.3111	0.0260
		A2. Facility and space planning	0.1674	0.0537	A2.1. Living quarters	0.2352	0.0126
			0.1674	0.0537	A2.2. Palliative care–related rooms	0.2321	0.0125
			0.1674	0.0537	A2.3. Other supporting facilities	0.1402	0.0075
			0.1674	0.0537	A2.4. Corridor layout	0.1786	0.0096
			0.1674	0.0537	A2.5. Safety settings	0.2140	0.0115
		A3. Supplies and equipment management	0.1750	0.0561	A3.1. Basic material provision and management	0.3549	0.0199
			0.1750	0.0561	A3.2. Transfer equipment provision and management	0.2925	0.0164
			0.1750	0.0561	A3.3. Medication use and management	0.3525	0.0198
		A4. Organizational management	0.2002	0.0642	A4.1. Organizational structure	0.4618	0.0297
			0.2002	0.0642	A4.2. Management systems	0.5382	0.0346
		A5. Financial support	0.1969	0.0632	A5.1. Financial support and management	1.0000	0.0632

B. Process indicators	0.4114	B1. Palliative care admission assessment service	0.0920	0.0378	B1.1. Admission criteria	0.4611	0.0175
			0.0920	0.0378	B1.2. Basic data collection and comprehensive assessment	0.5389	0.0204
		B2. Comfort care services	0.2092	0.0861	B2.1. Basic nursing services	0.5405	0.0465
			0.2092	0.0861	B2.2. Catheter care	0.4595	0.0395
		B3. Symptom management services	0.2071	0.0852	B3.1. Symptom assessment and management	1.0000	0.0852
		B4. Death education services	0.1317	0.0542	B4.1. Death education content	0.5816	0.0315
			0.1317	0.0542	B4.2. Methods and approaches to death education	0.4184	0.0227
		B5. Psychological support and humanistic care	0.1490	0.0613	B5.1. Communication with older residents and their families	0.2673	0.0164
			0.1490	0.0613	B5.2. Psychological support and humanistic care for older residents and their families	0.2868	0.0176
			0.1490	0.0613	B5.3. Protection of rights	0.2434	0.0149
			0.1490	0.0613	B5.4. Social support services	0.2026	0.0124
		B6. Post‐death arrangements and bereavement care services	0.1068	0.0439	B6.1. Post‐death arrangement services	0.5163	0.0227
			0.1068	0.0439	B6.2. Bereavement care services	0.4837	0.0213
		B7. Palliative care transfer services	0.1042	0.0429	B7.1. Transfer services and management	1.0000	0.0429

C. Outcome indicators	0.2678	C1. Comfort care outcomes	0.1495	0.0400	C1.1. Quality of basic nursing	0.5432	0.0217
			0.1495	0.0400	C1.2. Quality of catheter care	0.4568	0.0183
		C2. Quality of symptom management	0.1635	0.0438	C2.1. Quality of symptom assessment and management	1.0000	0.0438
		C3. Quality of death education	0.1052	0.0282	C3.1. Quality of death education for older residents	0.5889	0.0166
			0.1052	0.0282	C3.2. Quality of death education for family members	0.4111	0.0116
		C4. Quality of psychological support and humanistic care	0.1155	0.0309	C4.1. Quality of communication with older residents and their families	0.3346	0.0103
			0.1155	0.0309	C4.2. Quality of psychological support and humanistic care for older residents and their families	0.3775	0.0117
			0.1155	0.0309	C4.3. Status of rights protection and social support	0.2879	0.0089
		C5. Outcomes of post‐death arrangements and bereavement care	0.0803	0.0215	C5.1. Quality of post‐death arrangements	0.4944	0.0106
			0.0803	0.0215	C5.2. Quality of bereavement care services	0.5056	0.0109
		C6. Palliative care transfer service outcomes	0.0818	0.0219	C6.1. Quality of transfer services and management	1.0000	0.0219
		C7. Incidence of adverse events	0.0779	0.0209	C7.1. Incidence of falls/bed falls, pressure injuries, unplanned extubation, and other adverse events	1.0000	0.0209
		C8. Infection control quality	0.0800	0.0214	C8.1. Healthcare‐associated infection status	0.3549	0.0076
			0.0800	0.0214	C8.2. Implementation of healthcare‐associated infection prevention measures	0.2925	0.0063
			0.0800	0.0214	C8.3. Communicable disease prevention status	0.3525	0.0076
		C9. Documentation management	0.0789	0.0211	C9.1. Quality of medical care documentation	1.0000	0.0211
		C10. Feedback and quality improvement	0.0674	0.0180	C10.1. Feedback from older residents and their families	0.3985	0.0072
			0.0674	0.0180	C10.2. Staff feedback	0.2806	0.0051
			0.0674	0.0180	C10.3. Institutional quality improvement	0.3209	0.0058

## 4. Discussion

This study developed the final palliative care service quality assessment index system for Chinese nursing homes. On the basis of Donabedian’s “structure–process–outcome” framework, the system comprises 3 first‐level indicators, 22 second‐level indicators, and 47 third‐level indicators. Three rounds of Delphi expert consultation achieved a strong consensus, as evidenced by high questionnaire response rates (85.19%–88.57%), a high expert authority coefficient (> 0.859), and a significant increase in Kendall’s W from 0.189 to 0.315. The AHP confirmed strong internal consistency in the weight assignments (CR < 0.1). This index system offers an operational application of the Donabedian model in Chinese nursing homes and, through weight analysis, suggests potential priority areas for quality improvement.

The findings indicate that the first‐level “structural indicators” had a weight coefficient of 0.3208. Within this dimension, “human resources planning and management” was identified as the most critical second‐level indicator (0.0836). This discovery aligns with Ma’s [16] emphasis on human resources in community palliative care research, underscoring the foundational role of human resource allocation in ensuring the quality of services. Furthermore, “financial support and management” serves as the highest weighted third‐level indicator (0.0632) under the structural dimension, highlighting the critical impact of sustainable funding on service quality. However, existing research indicates that nursing homes universally face insufficient funding when advancing palliative care services [38]. This suggests the need to establish more stable funding support mechanisms at the policy level, such as medical insurance payment reforms and dedicated fiscal allocations [38]. These policy measures should synergize with institutional‐level human resource development to collectively strengthen the service foundation. The optimization of structural indicators mainly involved the addition and modification of indicator operational definitions, with no indicator deletions. For example, requirements were added to the operational definition of the “palliative care–related rooms” indicator, specifying dedicated spaces such as hospice rooms, farewell rooms, and areas for overnight stays by family members. This revision responds to the lack of farewell spaces and insufficient palliative care specialization [9]. A key modification was integrating the process item “multidisciplinary team collaboration and communication” into the operational definition of the structural item “staffing and workforce management.” This shifted it from a service activity to an institutional resource requirement and provided a pathway to address the limited multidisciplinary involvement in Chinese nursing homes [10].

The first‐level “process indicators,” weighted at 0.4114, constitute the most significant evaluation dimension within the final index system, reflecting their core value in service quality assessment and continuous improvement. Among these indicators, the second‐level indicator “comfort care services” has the highest weight (0.0861), which aligns with the fundamental purpose of palliative care to alleviate suffering and enhance the quality of life for terminally ill patients [14]. Its subordinate third‐level indicator, “basic nursing services,” is the second‐highest weighted among all third‐level indicators (0.0465), reflecting the core demand for essential daily care among older residents at the end of life. As Sieber et al. [39] indicated, a loss of independent living capacity can induce loneliness and depressive symptoms, diminish self‐esteem, and thereby compromise quality of life. Therefore, strengthening basic nursing services constitutes not only a direct response to the physiological needs of older residents with declining independence but also holistic care that addresses their dignity and psychological well‐being. In terms of indicator streamlining, indicators with overly broad definitions or unclear service measures (e.g., “admission and discharge services”) were deleted, as was “medication use” due to overlap with a structural indicator. Through Delphi expert deliberation, the second‐level indicator “palliative care education services” was refined into the more targeted “death education services,” addressing China’s societal avoidance of death‐related topics and educational gaps [40]. Additionally, preliminary interviews revealed that when residents can no longer articulate their wishes, family members often assume decision‐making authority, a dynamic that undermines residents′ self‐determination. Echoing the focus of the quality indicators for palliative care (Q‐PAC) framework [41], this study prioritizes psychological, social, and spiritual needs through specific indicators such as “communication with older residents and their families” and “protection of rights.” These additions mandate the documentation and verification of residents′ preference expression, ensuring that humanistic care is systematically embedded in practice.

The first‐level “outcome indicators” had a weight of 0.2678. Within this dimension, the second‐level indicator “quality of symptom management” (0.0438) and the third‐level indicator “quality of symptom assessment and management” (0.0438) were the highest weighted items at their respective levels. This emphasis aligns closely with the designation of symptom control as a core practice in the palliative care practice guideline (2025 edition) [14]. Elderly individuals in the terminal phase frequently experience multiple symptoms, such as pain and fatigue, due to aging, multiple illnesses, and cancer. These symptoms not only cause physical suffering but also trigger negative psychological experiences in this population [42]. As emphasized by Kreher [43], effective symptom management constitutes a central component of end‐of‐life care. To this end, this study established a systematic quantitative evaluation framework. Within the operational definition of “quality of symptom assessment and management,” culturally adapted and validated assessment tools were systematically incorporated. These include the Chinese version of the Palliative Performance Scale (C‐PPS) [44], the Chinese version of the Numerical Rating Scale (C‐NRS) [45], and the Chinese version of the Edmonton Symptom Assessment System (C‐ESAS) [46]. Their integration enables the standardized assessment of physical function, pain, and other core symptoms in older residents. During Delphi consultations, indicators such as “quality of service accessibility” were deleted for failing statistical thresholds or conceptual clarity, while the overly broad “effectiveness of specialist nursing” was disaggregated into more specific outcome indicators. Furthermore, to address high‐risk incidents such as falls and pressure injuries among terminally ill older residents in nursing homes [47, 48], the original third‐level indicator “service user safety” was elevated to a second‐level indicator “incidence of adverse events,” under which specific subindicators (e.g., incidence of falls/bed falls, pressure injuries, and unplanned extubation) and their calculation formulas were established. Field et al. [49] and St Clair et al. [50] have confirmed that adverse event rates are core metrics for monitoring institutional service quality, supporting this revision’s necessity and practical value.

This study follows an evidence‐based methodology that underpins the development of quality assessment index systems, thereby helping to ensure that the resulting system is grounded in both solid evidence and a robust structure. The Delphi expert consultation synthesized insights from specialists across multiple regions and disciplines nationwide, whereas the AHP transformed qualitative consensus into quantifiable priority rankings. This approach may offer useful guidance for practical application and help mitigate the risk of “indicator overgeneralization.” In terms of content design, the index system covers three core dimensions: structure, process, and outcome. It seeks to address key challenges in palliative care services within Chinese nursing homes, incorporating practical needs such as multidisciplinary team collaboration, death education, and rights protection into the evaluation scope. Specific operational definitions are provided to support practical feasibility. Its practical efficacy and broad applicability, however, require further validation in real‐world settings to bridge the gap between tool development and routine application.

## 5. Relevance to Clinical Practice

The index system constructed in this study may hold significant guidance and practical value for palliative care in nursing homes. First, it provides a systematic quality management framework that could assist institutions in moving beyond traditional experience‐based management toward a closed‐loop system for continuous quality improvement. Second, the clearly defined weight assignments offer a potential scientific basis for optimizing resource allocation, enabling managers to prioritize limited human and material resources in areas most critical to service quality. Most importantly, it suggests a shift in care philosophy from a reactive, “disease‐and‐task–centered” model to a proactive, “older adult–centered” holistic care paradigm. This shift would require caregivers to integrate psychological support, spiritual care, and respect for residents’ rights into daily practice while addressing physical symptoms, thereby improving the quality of end‐of‐life care.

## 6. Limitations

This study has several limitations. First, this study has only completed theoretical construction and content validity testing of the indicators, without systematic validation of their measurement properties in real‐world settings, which is the principal limitation. Second, while the modified Delphi method used quantitative screening criteria and multiple rounds to enhance objectivity, it inherently involves subjectivity in expert selection and interpretation of qualitative feedback, which may influence the final indicator set. Third, the current indicators do not fully address palliative care needs of specialized elderly populations in nursing homes, such as those with dementia, mental health conditions, or specific cultural backgrounds. Fourth, variations in healthcare systems and societal contexts may restrict international applicability, requiring local adaptation; meanwhile, regional disparities in resources and development stages within China may also need adaptive modifications or phased implementation in less developed areas. Future research should prioritize multicenter empirical studies to validate the feasibility, reliability, validity, and sensitivity of the index system across diverse care settings. In addition, complementary approaches (e.g., consensus conferences and multiple independent expert panels) should be used to cross‐validate the indicator set and mitigate the inherent subjectivity of the Delphi method. Supplementary indicators targeting residents with dementia, psychiatric conditions, and cultural diversity will be developed. The index system will be adapted and tested across different regional and cultural contexts, with phased implementation strategies for less developed areas. Ultimately, this will contribute to establishing a palliative care service quality assessment index system that combines dynamic adaptability and broad applicability.

## 7. Conclusion

This study developed a quality assessment index system for palliative care services tailored to Chinese nursing homes. Grounded in the actual conditions and service needs of these institutions, the system is built upon a strong foundation of expert consensus and scientific weight allocation. This study provides a preliminary scientific basis for self‐assessment and quality improvement efforts in nursing homes. However, the index system should undergo empirical field validation before being considered for regulatory or policy use. Future efforts should focus on refining this system through multicenter studies to promote its practical application, thereby enhancing the standardization and quality of palliative care services in Chinese nursing homes.

## Author Contributions

Lu Zhang and Xiao‐Qin Wu equally contributed to this work. Lu Zhang: conceptualization, methodology, project administration, investigation, formal analysis, and writing–original draft. Xiao‐Qin Wu: project administration, investigation, data curation, formal analysis, and writing–original draft. Ling Tan: investigation, data curation, and formal analysis. Ping‐Pin Wen: investigation and data curation. Zhi‐Xiang Sun: investigation and data curation. Mei‐Fang Yang: investigation and funding acquisition. Yue‐Lin Huang: conceptualization, resources, project administration, supervision, funding acquisition, and writing–review and editing. Jing Fu: conceptualization, methodology, supervision, funding acquisition, and writing–review and editing.

## Funding

This work was supported by the 2023 Science and Technology Programme of the Luzhou Bureau of Science, Technology, and Talent Work (grant number: 2023SYF117); the 2025 Annual Scientific Research Project of the Sichuan Hospital Management and Development Research Centre (grant number: SCYG2025‐28); and the Strategic Cooperation Project between the Luzhou City Government and Southwest Medical University (grant number: 2024LZXNYDJ100).

## Disclosure

All authors reviewed the manuscript and agreed to submit it for consideration for publication. The funders had no role in the study design, data analysis, decision to publish, or preparation of the manuscript.

## Ethics Statement

This study was conducted in accordance with the Declaration of Helsinki and approved by the Ethics Review Committee of Yibin Second People’s Hospital (Approval No. 2023‐220‐01). All participants voluntarily enrolled in the study, and each participant signed an informed consent form prior to the commencement of the research.

## Conflicts of Interest

The authors declare no conflicts of interest.

## Supporting Information

Additional supporting information can be found online in the Supporting Information section.

## Supporting information


**Supporting Information 1** Initial item pool of the quality assessment index system derived from the literature review.

**Supporting Information 2** Semistructured interview guides for facility administrators, professional caregivers, older residents, and their family members.

**Supporting Information 3** Demographics of interview respondents and summary of findings from semistructured interviews.

**Supporting Information 4** Detailed results of the third‐round Delphi expert consultation.

**Supporting Information 5** Complete list of finalized indicators in the palliative care quality assessment index system.

## Data Availability

Data supporting the findings of this study are available from the corresponding authors upon reasonable request.
