# Supplementary material for: Development of a Quality Assessment Index System for Palliative Care Services in Chinese Nursing Homes: A Modified Delphi and Analytic Hierarchy Process Study
Source: J Nurs Manag. 2026 Jul 6;2026:6031056. doi: 10.1155/jonm/6031056 (PMC13338572; doi:10.1155/jonm/6031056)
Supplement: Supplementary file 1 — Supporting Information 1 Initial item pool of the quality assessment index system derived from the literature review. [file JONM-2026-6031056-s002.docx]

Initial Item Pool for the Quality Assessment Index System for Palliative Care Services in Chinese Nursing Homes

| **First-level Indicator** | **Second-level Indicators** | **Third-level Indicators** | **Operational Definition** |
| --- | --- | --- | --- |
| Structural Evaluation | Personnel  Status | Personnel  Staffing | Doctors: 1. One licensed physician per 20 Older Residents; 2. One licensed physician per 10 palliative care beds Nurses: Two registered nurses per 20 Older Residents Care Attendants: 1. Staffing ratio of 1:4–1:6 per bed or allocated according to care level; 2. Nurse-to-attendant ratio of 1:3 Pharmacist: Deployed as required Psychological Counsellors: Provided as required Nutritionist: Provided as required Social workers: 1. At least one; 2. Deployed as required Volunteers: Deployed as required Other: As required |
|  |  | Staff Professional Competence | Proportion of personnel who have passed the assessment of professional competence in palliative care knowledge and skills |
|  |  | Training and Management | Palliative care service personnel training management records are standardised, with comprehensive training plans and implementation details (e.g., pre-service training, continuing education, on-the-job training content and duration) |
|  | Facilities and Room Configuration | Provision of care rooms and private rooms | 1. Provision of relatively independent end-of-life care rooms, farewell rooms, etc.; 2. Provision of facilities and supplies for overnight stays by relatives; 3. Rooms for end-of-life care recipients maintain suitable temperature and humidity with humanistic care considerations; 4. Room configuration and facilities for end-of-life care recipients (e.g., privacy curtains, emergency call systems, audio-visual playback equipment or systems) |
|  |  | Other  Additional  Layout | Provision of catering facilities, laundry rooms, offices, daily activity areas, and service support zones |
|  |  | Facility  Access  Layout | Scientific and rational design of pedestrian and goods access routes (e.g., dedicated passage for transporting remains) |
|  |  | Safety  Signage  Installation | Floor plan evacuation diagrams, firefighting and emergency equipment location signs, and other emergency directional signage |
|  | Equipment and Material Provision and Management | Basic  Supplies  Equipment | 1. Provision of oxygen facilities, suction equipment, decompression mattresses, pulse oximeters, nursing trolleys, etc.; 2. Equipment for rehabilitation, physiotherapy, psychological intervention, etc. |
|  |  | Transportation  Equipment | Availability of transport vehicles, shuttle buses, etc. |
|  |  | Supplies  and facilities  Equipment Management  Management | 1. Management systems, usage records, and maintenance status of supplies and equipment; 2. Management of medications brought by service users or delivered by relatives, and administration of medication to service users |
|  | Organizational Systems and Management | Organisation  Structure | Palliative care organizational structure, clear job responsibilities and service items |
|  |  | Management  Systems | 1. A robust system of management protocols for palliative care service delivery (e.g., service agreements, patient identification systems, medical record archiving, shift handover procedures); 2. Comprehensive safety management and emergency response protocols (e.g., fire safety, psychological crisis management); 3. Possession of service process regulations and quality control standards for palliative care (e.g., oral care protocols, pressure ulcer care protocols), with relevant services compliant with regulatory documentation |
|  |  | Funding  Support  and Management  Management | 1. Adequate financial support for palliative care services; 2. Standardised management system for palliative care financial receipts and expenditures |
| Process Evaluation | Service delivery | Admission and Discharge Services | Admission and discharge assessments (e.g., nutritional screening), collection of basic client information (e.g., personal details, social relationships, medical history), and processing of admission and discharge procedures |
|  |  | Basic  Nursing  Services | 1. Documentation of the provision of assisted bathing, oral care, skin care, and positioning care services according to the service user's capabilities; 2. Documentation of procedures involving various tubes (e.g., urinary catheters, enteral feeding tubes), pressure ulcers/wounds/stomas, and vital signs monitoring; 3. Documentation of periodic assessments and formulation of personalised nutritional and fluid replacement plans; records of nasogastric feeding or parenteral nutrition administration for older residents unable to feed independently |
|  |  | Symptoms  Assessment  and  Management | 1. Regular dynamic assessment of symptoms (e.g., pain, dyspnoea) using standardised tools, timely intervention, and documentation of symptom control implementation; 2. Assessment, observation, nursing implementation, and documentation of terminal symptoms (e.g., delirium, clammy skin, stridor) |
|  |  | Medication Use | The provision and types of opioid medications administered, alongside the provision, types, and management of psychotropic sedatives |
|  |  | Multidisciplinary Team Collaboration and Communication | 1. Establishment of multidisciplinary teams (e.g., social workers, volunteers, clinical nursing specialists, speech pathologists, psychologists, physicians, etc.); 2. Multidisciplinary team collaboration and consultation arrangements |
|  |  | Continuity of care | Implementation of continuity of care for service users |
|  |  | Psychological and Spiritual Support for Service Users | 1. Documentation of assessments, management, and emotional support for service users' psychological conditions (e.g., anxiety, depression); 2. Regular assessment and documentation of service users' end-of-life preferences (e.g., preferred place of death), palliative care treatment choices, or expectations; 3. Documentation of assessments of service users' spiritual needs and spiritual distress, along with implementation records reflecting their religious beliefs and cultural customs; 4. Guidance for service users in formulating realistic and achievable wishes, completing unfinished matters, and documentation of implementation outcomes |
|  |  | Psychological and Spiritual Support for Relatives | 1. Documentation of relatives' psychological state (e.g., grief, stress), needs, and management implementation, alongside provision of emotional and spiritual support; 2. Whether visiting hours for relatives of terminally ill service recipients are unrestricted |
|  |  | Communication with Service Users and Relatives | 1. Implementation of assisting in establishing advance healthcare directives with family involvement, and conveying the service user's wishes to relatives; 2. Regular updates to relatives regarding the service user's condition using appropriate methods, while respecting family privacy; facilitating communication to support service user and family decision-making; 3. Facilitating family members' compassionate companionship and open communication with the service user, and managing family dynamics |
|  |  | Social support services | 1. Seek social support (e.g., social and policy assistance) tailored to the service user's circumstances; 2. Safeguarding the service user's rights to medical coverage, social welfare benefits, and coordinating access to social resources (e.g., legal advice, funeral services, post-mortem arrangements); |
|  |  | Palliative Care Education | Conducting palliative care education for service recipients and their relatives (e.g., science communication on end-of-life scenarios, life education, etc.) |
|  |  | Funeral Arrangements and Bereavement Support Services | 1. Death registration, body preparation (including mortuary care, terminal disinfection of the residence, and identification procedures), transportation, and encouraging relatives to participate in body preparation; 2. Advance guidance for relatives in preparing for post-death arrangements (e.g., burial garments, funeral arrangements, etc.); 3. Implementation of comprehensive bereavement care and grief counselling programmes (e.g., assessment of bereavement risks and needs, regular follow-ups), alongside provision of bereavement services and grief support |
|  | Respect in Service Delivery | Respect for service users and relatives | 1. Whether communication with service users and relatives respects cultural diversity; 2. Whether end-of-life conversations are facilitated for service users; 3. Implementation of personalised care respecting service users' and relatives' preferences, religious beliefs, wishes (e.g., final wishes), privacy, etc. 4. Whether arrangements for final rites respect the wishes and customs of service recipients and their relatives |
|  | Service Accessibility | Availability of services during rest periods | Availability of night-time and weekend services |
|  |  | Availability of consultation services | Whether palliative care counselling is provided for service recipients and their relatives (e.g., admission criteria, service offerings, psychological counselling, bereavement support, etc.) |
| Outcome Evaluation | Daily care provision | Service User Safety | Incidence of adverse events such as falls, bed falls, suicide attempts, medication errors, and unintended tube removal |
|  |  | Basic nursing care | 1. Incidence of high-risk pressure ulcers (Stages II-IV) or pressure ulcers of unclear staging; 2. Nutritional status; 3. Incidence of nosocomial infections |
|  | Effectiveness of specialist nursing | Symptom management status | End-of-life symptom control (pain score < 3, depression, dyspnoea, delirium, etc.) |
|  |  | Psychological and spiritual support provision | 1. Provision of personalised support to service users (as assessed via the Consumer Quality of Care for Palliative Care (CQ) Scale or other methods: number of service users reporting emotional support / total number of service users × 100%);  2. Personalised support provided to relatives (as assessed via the Consumer Quality of Care (CQ) Scale or other methods: number of relatives receiving emotional support / total number of service recipients and relatives × 100%);  3. Prevalence of depression among service users;  4. Prevalence of depression among relatives;  5. Percentage of service users with documented end-of-life care preferences |
|  |  | Respect for Service Users and Relatives | 1. Respect for service users and relatives (number of service users and relatives who felt respected / total number of service users and relatives × 100%); 2. Proportion of service users who died at their preferred end-of-life location |
|  |  | Communication with service users and relatives | 1. Communication between multidisciplinary teams and service users and/or relatives (Number of service users and/or relatives who communicated with medical team members / Total number of service users and relatives × 100%); 2. Shared decision-making (Number of service users whose decisions involved relatives/Total number of deceased service users × 100%); 3. Acceptance of palliative care documentation (e.g., advance care planning) (Number of service users with palliative care documentation / Total number of deceased service users × 100%) |
|  |  | Overall care provision | 1. Quality of death compliance rate (as assessed by the QOD-LTC scale or other means: number of service users meeting quality of death standards / total number of deceased service users × 100%);  2. End-of-Life Care Quality Compliance Rate (as assessed by the EOLD scale or other methods: number of service users meeting end-of-life care quality standards / total number of deceased service users × 100%);  3. Quality of life status (using relevant quality of life scales, e.g. the QUALID Quality of Life Scale);  4. In-facility mortality rate (number of service users dying within the nursing home / total number of deceased service users × 100%) and 90-day terminal hospitalisation rate (number of service users hospitalised within 90 days of death / total number of deceased service users × 100%) |
|  | Records and Management | Medical Documentation Recording and Management | Key Content Recording and Management Compliance with standard protocols (e.g., end-of-life decision-making communication, medical condition documentation, resuscitation records), adhering to medical documentation writing standards |
|  |  | Recording and Management of Other Documents | Establishment and completion standards for documentation agreements, along with their consolidated management practices |
|  | Evaluation and Improvement | Feedback from Service Recipients and Relatives | Service satisfaction evaluations and complaint rates from service recipients and relatives |
|  |  | Staff Feedback | Staff self-assessment and work recommendations |
|  |  | Institutional Quality Improvement | Institutional periodic spot checks and assessments, quality improvement initiatives |

Note: CQ Scale, Consumer Quality Index, assessing the care experience of patients and their families during end-of-life care; QOD-LTC Scale, Quality of Dying in Long-Term Care, assessing the psychosocial quality of dying for individuals who pass away in long-term care settings such as nursing homes; EOLD Scale, End-of-Life in Dementia, measuring physical comfort and distress symptoms in terminally ill dementia patients; QUALID Scale, Quality of Life in Late-Stage Dementia, assessing the quality of life for individuals with severe dementia.
