# Supplementary material for: Development of a Quality Assessment Index System for Palliative Care Services in Chinese Nursing Homes: A Modified Delphi and Analytic Hierarchy Process Study
Source: J Nurs Manag. 2026 Jul 6;2026:6031056. doi: 10.1155/jonm/6031056 (PMC13338572; doi:10.1155/jonm/6031056)
Supplement: Supplementary file 2 — Supporting Information 2 Semistructured interview guides for facility administrators, professional caregivers, older residents, and their family members. [file JONM-2026-6031056-s003.docx]

**Semi-Structured Interview Guides**

Separate semi-structured interview guides were developed for three stakeholder groups: facility administrators, professional caregivers (medical staff and nursing aides), and older residents together with their family members.

**1. For Facility Administrators**

*1.1.* What specific palliative care services are currently being provided in your nursing home?

*1.2.* What difficulties have you encountered during the management of palliative care services?

*1.3.* What problems do you perceive currently exist in the management of palliative care services?

*1.4.* What particular aspects do you pay special attention to during the management of palliative care services?

*1.5.* What content do you believe should be included in the quality assessment of palliative care services in nursing homes?

*1.6.* Please share your feelings or experiences regarding the implementation or management of palliative care services.

**2. For Professional Caregivers (Medical Staff and Nursing Assistants)**

*2.1.* What specific palliative care tasks are currently included in your daily work?

*2.2.* What deficiencies do you perceive in the current palliative care services you provide?

*2.3.* What difficulties or problems have you encountered during the delivery of palliative care services?

*2.4.* What content do you believe should be included in the quality assessment of palliative care services in nursing homes?

*2.5.* Please share your feelings or experiences regarding the implementation or management of palliative care services.

**3. For Older Residents and Their Family Members**

*3.1.* What palliative care services have you or your family member currently received?

*3.2.* Are you familiar with palliative care? If yes, from what sources have you learned about it?

*3.3.* Do you think palliative care services in nursing homes are easily accessible?

*3.4.* What aspects of the palliative care services you or your family member have received are you satisfied with?

*3.5.* What content do you believe a satisfactory palliative care service should include?

*3.6.* Are you willing to receive palliative care services in a nursing home for yourself or to have your family member receive them? Why or why not?
