# Supplementary material for: Development of a Quality Assessment Index System for Palliative Care Services in Chinese Nursing Homes: A Modified Delphi and Analytic Hierarchy Process Study
Source: J Nurs Manag. 2026 Jul 6;2026:6031056. doi: 10.1155/jonm/6031056 (PMC13338572; doi:10.1155/jonm/6031056)
Supplement: Supplementary file 3 — Supporting Information 3 Demographics of interview respondents and summary of findings from semistructured interviews. [file JONM-2026-6031056-s004.docx]

**Respondent Demographics and Interview Findings**

**1. Basic Information of Respondents**

This study ultimately included 25 interviewees: five nursing home managers, all female, aged 43–58 years, with over eight years of experience in nursing home management and over four years in palliative care management; thirteen professional care staff, comprising four doctors, six nurses, and three nursing assistants. There were three males and ten females, aged 24 to 59 years. All had over two years of experience in elderly care or medical work, and at least two years of experience in palliative care. Three older residents and four family members participated in the study. Four were male and three female, aged 50 to 79 years. Family members included one spouse and three children. Basic demographic data of research subjects are presented in Tables 1–3.

TABLE 1: Basic Information of Nursing Home Managers (n=5).

| **ID** | **Age** | **Gender** | **Educational Background** | **Professional Title** | **Years of Experience in Nursing Home Management** | **Years of Experience in Palliative Care Management** |
| --- | --- | --- | --- | --- | --- | --- |
| A1 | 58 | Female | Bachelor's Degree | Senior Professional | 18 | 4 |
| A2 | 55 | Female | Bachelor's Degree | Senior Professional | 24 | 12 |
| A3 | 54 | Female | Bachelor's Degree | Senior Professional | 15 | 5 |
| A4 | 46 | Female | Bachelor's Degree | Associate Professor | 10 | 7 |
| A5 | 43 | Female | Bachelor's Degree | Associate Professor | 8 | 4 |

TABLE 2: Basic Information of Professional Care Staff (Medical Personnel and Nursing Assistants) (n=13).

| **ID** | **Age** | **Gender** | **Educational Background** | **Professional Title** | **Years of Experience in Elderly Care or Medical Work** | **Years of Experience in Palliative Care** |
| --- | --- | --- | --- | --- | --- | --- |
| B1 | 54 | Female | Bachelor's Degree | Chief Physician | 20 | 6 |
| B2 | 35 | Female | Bachelor's Degree | Attending Physician | 7 | 3 |
| B3 | 31 | Male | Bachelor's Degree | Rehabilitation Therapist | 6 | 4 |
| B4 | 28 | Male | Bachelor's Degree | Physician | 5 | 2 |
| C1 | 49 | Female | Bachelor's Degree | Deputy Head Nurse | 20 | 10 |
| C2 | 39 | Female | Bachelor's Degree | Senior Nurse | 15 | 4 |
| C3 | 37 | Female | Bachelor's Degree | Senior Nurse | 8 | 5 |
| C4 | 36 | Female | Bachelor's Degree | Senior Nurse | 10 | 3 |
| C5 | 27 | Female | Specialist | Nurse | 6 | 3 |
| C6 | 24 | Female | Specialist | Nurse | 3 | 2 |
| D1 | 59 | Male | Junior High School | None | 5 | 3 |
| D2 | 56 | Female | Junior High School | None | 2 | 2 |
| D3 | 55 | Female | Junior High School | None | 3 | 2 |

**TABLE 3: Basic Information of older residents and Their Families (n=7).**

| **ID** | **Age** | **Gender** | **Educational Attainment** | **Occupation** | **Relationship to the Elderly Resident** |
| --- | --- | --- | --- | --- | --- |
| E1 | 79 | Male | Technical Secondary School | Retired Employee | Self |
| E2 | 75 | Male | Junior Secondary Education | Self-Employed | Self |
| E3 | 69 | Female | Primary School | Self-Employed | Self |
| F1 | 73 | Male | Technical Secondary School | Retired Employee | Spouse |
| F2 | 58 | Female | Technical Secondary School | Retired Employee | Daughter |
| F3 | 51 | Male | Secondary School | Worker | Son |
| F4 | 50 | Female | Undergraduate | Teacher | Daughter |

**2. Analysis of Interview Themes**

Through transcription, coding, summarization, and verification, seven preliminary quality assessment indicators were identified.

*2.1. Financial Support.* Funding constraints impose dual limitations on palliative care provision within nursing homes. At the governmental level, unclear funding restricts investment; at the market level, exclusion from insurance and low willingness to pay diminish incentives for private nursing homes. A1: "Currently, there is no dedicated government funding for palliative care development. Even where support exists, it prioritizes public hospitals. Fundamentally, the greatest obstacles to implementing palliative care are funding and national policy issues, including medical insurance and social security coverage. If these could be resolved, introducing palliative care within our nursing homes would be far less challenging". A2: "We charge for palliative care on a daily basis, meaning families cannot claim medical insurance reimbursement. For instance, if a family member requires medical treatment, they will consider public hospitals as an alternative. Consequently, although we have established palliative care wards, we receive relatively few older residents requiring such services". B1: "It's currently challenging to develop palliative care here, primarily because we are effectively operating at a loss for each patient we serve. Revenue-wise, it's simply unsustainable, which may lead to certain services being discontinued".

*2.2. Staff Qualifications and Training Management.* Admission standards for palliative care staff remain undefined, and turnover is high. Nursing assistants tend to be older, less educated, and lack access to comprehensive training. A4: "Our care staff are predominantly older, mostly around 50 years old, with limited education. Given the low pay, younger individuals are unwilling to enter this role, making training this demographic particularly challenging". D1: "Many people are reluctant to become nursing assistants, perceiving it as socially undesirable. Only those of us who are older and struggle to find other work end up in this role". C4: "All our nursing staff undergo a one-month probationary period. Only those capable of working shifts independently are offered contracts; those who fail to meet this requirement are dismissed. Further training is required after induction. Training our carers remains challenging. With few nurses available, tasks such as monitoring blood sugar and blood pressure often fall to them. Their low educational attainment makes this aspect of training particularly frustrating". C6: "Some of our carers have pursued nursing qualifications. Those with certificates receive slightly higher pay, though certification isn't strictly required".

*2.3. Satisfaction.* Satisfaction surveys serve as crucial benchmarks for optimizing palliative care quality, yet their practical application remains significantly constrained. Current surveys predominantly target family members, as most older residents lack the capacity for self-expression. As C4 noted: "We conduct annual satisfaction surveys with families, as the majority of older residents cannot communicate their views. These results inform our service improvements". Furthermore, some surveys are not effectively implemented. Limited medical staffing and professional capacity directly impact service experiences, leading to dissatisfaction. A2 noted: "We also conduct satisfaction surveys, but sometimes they are not reviewed. High staff turnover and shortages mean that our doctors are retiring, resulting in inadequate medical capabilities. These factors contribute to dissatisfaction among older residents and their families".

*2.4. Symptom Management.* Symptom management has gained recognition among nursing home staff, yet the absence of in-house medical professionals necessitates collaboration with external healthcare providers. Concurrently, the lack of specialized personnel results in suboptimal symptom management standards. C4 explained: "Our nursing home doesn't actually have doctors; we rely on physicians from the adjacent medical institution to assist us. For instance, when pain management or intravenous therapy is required, their doctors handle these procedures for us. However, for psychological symptoms, we cannot provide specialized counselling as we lack qualified psychotherapists". E1, F2, and F4 all expressed: "Seeing older residents suffering in bed is distressing. Knowing the illness is incurable, we eventually accept it over time. Our hope is simply that they experience no suffering while alive and pass away peacefully".

*2.5. Adverse Events.* Safety is a primary concern for all care facilities. C4: "In our facility, the most common incidents are falls, bed falls, and pressure ulcers, with occasional scalding injuries. Therefore, we must pay particular attention to these areas".

*2.6. Palliative Care Education for Families and Residents.* Educating families and residents about palliative care is crucial for improving service acceptance; however, it faces dual challenges, including cultural attitudes and professional limitations. On the one hand, traditional beliefs have a profound influence on end-of-life choices. B2 noted: "Many elderly wish to return home before passing, yet families often resist them dying at home". This conflict directly hinders the implementation of palliative care. On the other hand, education itself suffers from a lack of expertise and communication barriers. B2 candidly admits, "Our palliative care education is unprofessional and ineffective". Simultaneously, C3 notes: "Families and older residents are reluctant to discuss death, making communication extremely difficult". This renders education efforts ineffective in reaching and altering audience perceptions.

2.7. Improving Palliative Care Service Systems. Most nursing homes lack dedicated palliative care service systems, resulting in inconsistent service standards and suboptimal quality. A3 stated: "We have no specific palliative care protocols. When an elderly person arrives, we provide care according to our facility's standard procedures, primarily focusing on daily living support and symptom management".
