# Supplementary material for: Development of a Quality Assessment Index System for Palliative Care Services in Chinese Nursing Homes: A Modified Delphi and Analytic Hierarchy Process Study
Source: J Nurs Manag. 2026 Jul 6;2026:6031056. doi: 10.1155/jonm/6031056 (PMC13338572; doi:10.1155/jonm/6031056)
Supplement: Supplementary file 4 — Supporting Information 4 Detailed results of the third‐round Delphi expert consultation. [file JONM-2026-6031056-s005.docx]

Results of the Third-Round Delphi Consultation for the Finalized Indicators

| **First-level Indicators** | **Second-level Indicators** | **Third-level Indicators** | **Mean** | **Standard Deviation** | **Coefficient of Variation** | **Full Score Rate (%)** |
| --- | --- | --- | --- | --- | --- | --- |
| A Structural Indicators |  |  | 4.652 | 0.573 | 0.123 | 69.57 |
|  | A1 Human Resources Planning and Management |  | 5.000 | 0.000 | 0.000 | 100.00 |
|  |  | A1.1 Staffing and Workforce Management | 4.783 | 0.518 | 0.108 | 82.61 |
|  |  | A1.2 Personnel Qualification Requirements | 4.348 | 0.714 | 0.164 | 47.83 |
|  |  | A1.3 Training and Management | 4.565 | 0.662 | 0.145 | 65.22 |
|  | A2 Facility and Space Planning |  | 4.435 | 0.728 | 0.164 | 56.52 |
|  |  | A2.1 Living Quarters | 4.304 | 0.765 | 0.178 | 47.83 |
|  |  | A2.2 Palliative Care-Related Rooms | 4.348 | 0.775 | 0.178 | 52.17 |
|  |  | A2.3 Other Supporting Facilities | 4.261 | 0.689 | 0.162 | 39.13 |
|  |  | A2.4 Corridor Layout | 4.304 | 0.822 | 0.191 | 52.17 |
|  |  | A2.5 Safety Settings | 4.826 | 0.388 | 0.080 | 82.61 |
|  | A3 Supplies and Equipment Management |  | 4.435 | 0.662 | 0.149 | 52.17 |
|  |  | A3.1 Basic Material Provision and Management | 4.391 | 0.783 | 0.178 | 56.52 |
|  |  | A3.2 Transfer Equipment Provision and Management | 4.522 | 0.665 | 0.147 | 60.87 |
|  |  | A3.3 Medication Use and Management | 4.870 | 0.344 | 0.071 | 86.96 |
|  | A4 Organizational Management |  | 4.870 | 0.458 | 0.094 | 91.30 |
|  |  | A4.1 Organizational Structure | 4.348 | 0.775 | 0.178 | 52.17 |
|  |  | A4.2 Management Systems | 4.304 | 0.822 | 0.191 | 52.17 |
|  | A5 Financial Support |  | 4.913 | 0.288 | 0.059 | 91.30 |
|  |  | A5.1 Financial Support and Management | 4.435 | 0.788 | 0.178 | 60.87 |
| B Process Indicators |  |  | 4.739 | 0.541 | 0.114 | 78.26 |
|  | B1 Palliative Care Admission Assessment Service |  | 4.826 | 0.491 | 0.102 | 86.96 |
|  |  | B1.1 Admission Criteria | 4.391 | 0.783 | 0.178 | 56.52 |
|  |  | B1.2 Basic Data Collection and Comprehensive Assessment | 4.391 | 0.722 | 0.164 | 52.17 |
|  | B2 Comfort Care Services |  | 4.957 | 0.209 | 0.042 | 95.65 |
|  |  | B2.1 Basic Nursing Services | 4.478 | 0.665 | 0.149 | 56.52 |
|  |  | B2.2 Catheter Care | 5.000 | 0.000 | 0.000 | 100.00 |
|  | B3 Symptom Management Services |  | 4.913 | 0.288 | 0.059 | 91.30 |
|  |  | B3.1 Symptom Assessment and Management | 4.913 | 0.288 | 0.059 | 91.30 |
|  | B4 Death Education Services |  | 4.522 | 0.665 | 0.147 | 60.87 |
|  |  | B4.1 Death Education Content | 4.522 | 0.665 | 0.147 | 60.87 |
|  |  | B4.2 Methods and Approaches to Death Education | 4.870 | 0.344 | 0.071 | 86.96 |
|  | B5 Psychological Support and Humanistic Care |  | 4.435 | 0.728 | 0.164 | 56.52 |
|  |  | B5.1 Communication with Older Residents and their families | 4.870 | 0.344 | 0.071 | 86.96 |
|  |  | B5.2 Psychological Support and Humanistic Care for Older Residents and Their Families | 4.304 | 0.765 | 0.178 | 47.83 |
|  |  | B5.3 Protection of Rights | 4.870 | 0.344 | 0.071 | 86.96 |
|  |  | B5.4 Social Support Services | 4.174 | 0.778 | 0.186 | 39.13 |
|  | B6 Post-Death Arrangements and Bereavement Care Services |  | 4.261 | 0.752 | 0.176 | 43.48 |
|  |  | B6.1 Post-Death Arrangement Services | 4.522 | 0.511 | 0.113 | 52.17 |
|  |  | B6.2 Bereavement Care Services | 4.304 | 0.703 | 0.163 | 43.48 |
|  | B7 Palliative Care Transfer Services |  | 4.348 | 0.714 | 0.164 | 47.83 |
|  |  | B7.1 Transfer Services and Management | 4.348 | 0.647 | 0.149 | 43.48 |
| C Outcome Indicators |  |  | 4.957 | 0.209 | 0.042 | 95.65 |
|  | C1 Comfort Care Outcomes |  | 4.957 | 0.209 | 0.042 | 95.65 |
|  |  | C1.1 Quality of Basic Nursing | 4.957 | 0.209 | 0.042 | 95.65 |
|  |  | C1.2 Quality of Catheter Care | 4.913 | 0.288 | 0.059 | 91.30 |
|  | C2 Quality of Symptom Management |  | 4.913 | 0.288 | 0.059 | 91.30 |
|  |  | C2.1 Quality of Symptom Assessment and Management | 4.348 | 0.775 | 0.178 | 52.17 |
|  | C3 Quality of Death Education |  | 4.565 | 0.662 | 0.145 | 65.22 |
|  |  | C3.1 Quality of Death Education for Older Residents | 4.261 | 0.752 | 0.176 | 43.48 |
|  |  | C3.2 Quality of Death Education for Family Members | 4.391 | 0.783 | 0.178 | 56.52 |
|  | C4 Quality of Psychological Support and Humanistic Care |  | 4.609 | 0.722 | 0.157 | 73.91 |
|  |  | C4.1 Quality of Communication with Older Residents and Their Families | 4.652 | 0.487 | 0.105 | 65.22 |
|  |  | C4.2 Quality of Psychological Support and Humanistic Care for Older Residents and Their Families | 4.348 | 0.714 | 0.164 | 47.83 |
|  |  | C4.3 Status of Rights Protection and Social Support | 4.348 | 0.775 | 0.178 | 52.17 |
|  | C5 Outcomes of Post-Death Arrangements and Bereavement Care |  | 4.391 | 0.783 | 0.178 | 56.52 |
|  |  | C5.1 Quality of Post-Death Arrangements | 4.435 | 0.662 | 0.149 | 52.17 |
|  |  | C5.2 Quality of Bereavement Care Services | 4.217 | 0.671 | 0.159 | 34.78 |
|  | C6 Palliative Care Transfer Service Outcomes |  | 4.348 | 0.775 | 0.178 | 52.17 |
|  |  | C6.1 Quality of Transfer Services and Management | 4.304 | 0.765 | 0.178 | 47.83 |
|  | C7 Incidence of Adverse Events |  | 4.478 | 0.731 | 0.163 | 60.87 |
|  |  | C7.1 Incidence of Falls/Bed Falls, Pressure Injuries, Unplanned Extubation and Other Adverse Events | 4.913 | 0.288 | 0.059 | 91.30 |
|  | C8 Infection Control Quality |  | 4.739 | 0.541 | 0.114 | 78.26 |
|  |  | C8.1 Healthcare-Associated Infection Status | 4.870 | 0.344 | 0.071 | 86.96 |
|  |  | C8.2 Implementation of Healthcare-associated Infection Prevention Measures | 4.913 | 0.288 | 0.059 | 91.30 |
|  |  | C8.3 Prevention of Communicable Diseases | 4.870 | 0.344 | 0.071 | 86.96 |
|  | C9 Documentation Management |  | 4.348 | 0.775 | 0.178 | 52.17 |
|  |  | C9.1 Quality of Medical Care Documentation | 4.913 | 0.288 | 0.059 | 91.30 |
|  | C10 Feedback and Quality Improvement |  | 4.478 | 0.665 | 0.149 | 56.52 |
|  |  | C10.1 Feedback from Older Residents and their families | 4.870 | 0.344 | 0.071 | 86.96 |
|  |  | C10.2 Staff Feedback | 4.261 | 0.752 | 0.176 | 43.48 |
|  |  | C10.3 Institutional Quality Improvement | 4.261 | 0.752 | 0.176 | 43.48 |
