# Supplementary material for: Development of a Quality Assessment Index System for Palliative Care Services in Chinese Nursing Homes: A Modified Delphi and Analytic Hierarchy Process Study
Source: J Nurs Manag. 2026 Jul 6;2026:6031056. doi: 10.1155/jonm/6031056 (PMC13338572; doi:10.1155/jonm/6031056)
Supplement: Supplementary file 5 — Supporting Information 5 Complete list of finalized indicators in the palliative care quality assessment index system. [file JONM-2026-6031056-s001.docx]

Finalized Quality Assessment Index System for Palliative Care Services in Chinese Nursing Homes

| **First-level Indicators** | **Second-level Indicators** | **Third-level Indicators** | **Operational Definition** |
| --- | --- | --- | --- |
| A Structural Indicators | A1 Human Resources Planning and Management | A1.1 Staffing and Workforce Management | 1. Medical Staff: At least one Associate Chief Physician must be appointed. One Licensed Physician must be assigned per ten palliative care beds 2. Nurses: At least one senior nurse should be assigned, with three registered nurses per 20 palliative care beds 3. Care Workers: Staffing ratio of 1:4 to 1:6 per bed, or allocated according to care level 4. Pharmacists and Rehabilitation Therapists: Deployed as required; nursing homes with in-house nursing stations shall have at least one of each 5. Counsellors and Nutritionists: At least one of each should be provided 6. Social workers and volunteers: At least one of each should be assigned to the palliative care area/care room 7. Establish a comprehensive multidisciplinary team collaboration and communication management mechanism |
|  |  | A1.2 Personnel Qualification Requirements | 1. Directors and deputy directors of nursing homes must hold a college diploma or higher qualification 2. Management personnel responsible for palliative care services shall be individuals holding professional qualifications in medical or nursing practice 3. Clinical practitioners, registered nurses, social workers, counsellors and other technical professionals shall hold valid professional qualifications or practising certificates commensurate with their roles, possessing specialised knowledge and skills in elderly care services and palliative care 4. Care assistants shall commence duties only after completing pre-service training and achieving qualification 5. All staff providing personal care, dietary services, or medical nursing must hold valid health certificates |
|  |  | A1.3 Training and Management | 1. The facility manager must undergo professional training annually 2. Palliative care service personnel shall receive no fewer than two routine training sessions annually (covering professional ethics, theoretical knowledge, practical skills, etc.) and no fewer than four specialised training sessions related to palliative care 3. Establish an assessment and reward/penalty system for palliative care, standardise training management records, and refine training plans and implementation details |
|  | A2 Facility and Space Planning | A2.1 Living Quarters | 1. The average usable area per bed within a room shall not be less than 6m². The number of beds in a room shall not exceed four, with a minimum distance of 1 metre between beds. Privacy curtains shall be installed between beds, and sufficient space shall be provided beside beds for nursing and emergency procedures 2. Rooms shall have natural lighting and ventilation. Winter room temperature shall be ≥18°C, summer room temperature 26–28°C, humidity 50–60%, daytime noise level ≤50dB, night-time noise level ≤40dB 3. Rooms shall be equipped with en-suite lavatory and bathing facilities, featuring effective ventilation systems to eliminate odours 4. Furniture shall be appropriately sized and secure, incorporating barrier-free handrails and protective flooring designs compliant with current national standards |
|  |  | A2.2 Palliative Care-Related Rooms | 1. Facilities shall include hospice rooms (for daily living needs of terminally ill residents), consultation rooms, and farewell rooms, each with a usable area of no less than 15m². These spaces shall prioritise privacy and reflect compassionate care 2. Beyond meeting basic residential requirements, design elements such as natural light, artificial lighting, and colour schemes should be warm and comforting. Decorations may include fresh flowers, murals, calligraphy, photographs of loved ones, or images and personal items cherished by the patient 3. Facilities and supplies for overnight stays by accompanying family members shall be provided |
|  |  | A2.3 Other Supporting Facilities | 1. The provision of food preparation room, communal bathing facilities, communal laundry areas, offices, daily activity spaces, and service support zones shall comply with current relevant standards |
|  |  | A2.4 Corridor Layout | 1. Clear passage widths shall not be less than 1.40m, with continuous handrails installed on both sides at key locations 2. Floors, steps, stair treads, and ramps shall be surfaced with non-slip materials 3. Design pedestrian and goods access routes scientifically and rationally to ensure efficiency and unimpeded flow; provide dedicated routes for transporting mortal remains 4. Accessible lifts shall be provided on all floors above ground level, with at least one capable of accommodating flatbed trolleys or stretchers, separate from goods lifts |
|  |  | A2.5 Safety Settings | 1. Windows and doors in living quarters and activity areas shall incorporate safety measures and be equipped with adequate lighting to ensure older residents' safety during night-time activities 2. Indoor signage indicating boiling water, hot water, ultraviolet disinfection, fall detection, and bed exit should be provided. Emergency call buttons should be installed beside beds and in toilet facilities 3. Buildings shall incorporate buffer zones sufficient for safe evacuation. Floor plans shall display emergency directional signage including evacuation diagrams and indicators for firefighting and emergency equipment locations |
|  | A3 Supplies and Equipment Management | A3.1 Basic Material Provision and Management | 1. Medical equipment including oxygen concentrators, oxygen cylinders, nebulisers, suction devices, blood glucose monitors, pulse oximeters, sphygmomanometers, thermometers, fingertip pulse oximeters, and nursing trolleys shall be provided 2. Provide auxiliary equipment including pressure-relieving mattresses, bedside hair washers, seated bath chairs, commode chairs, wheelchairs, and patient transfer beds 3. Provide washing facilities including handwashing basins, disinfectant solutions, ultraviolet lamps, air disinfection units, and bed unit disinfectants 4. Comprehensive management protocols for material/equipment and item disinfection, with detailed and clear usage and maintenance records |
|  |  | A3.2 Transfer Equipment Provision and Management | 1. Equipped with transport vehicles, stretcher trolleys, wheelchairs and other transfer equipment, with comprehensive usage and maintenance protocols |
|  |  | A3.3 Medication Use and Management | 1. Understand older residents' medication regimens and establish a management system for medications brought by older residents and their families, with a 100% implementation rate 2. Adhere to individualized medication principles by developing and implementing standardized verification protocols (e.g., prescription and drug identity checks). Ensure medications are dispensed in-hand and administered under direct observation to prevent diversion or hoarding. 3. For cancer pain older residents, adhere to the WHO recommended three-step analgesic ladder: non-steroidal anti-inflammatory drugs (NSAIDs) → weak opioids → strong opioids. Opioids and psychotropic sedatives must be stored and administered in accordance with controlled substances regulations, ensuring accessibility of basic analgesics |
|  | A4 Organizational Management | A4.1 Organizational Structure | 1. The palliative care organizational structure, role responsibilities, and service provisions shall be clearly defined |
|  |  | A4.2 Management Systems | 1. A comprehensive system of palliative care service procedures is in place, including admission and discharge protocols, service agreements, and medical record archiving 2. Possesses operational protocols and quality control standards for palliative care services, including oral care, pressure injuries, pain management, and infection control 3. A robust safety management and emergency response system is in place, covering self-harm, falls, bed falls, choking, aspiration, scalding, psychological crisis intervention, and fire safety 4. Standardised service management protocols, including shift handover procedures, access and visiting arrangements for older residents and third parties, service quality satisfaction assessments, and complaint management systems |
|  | A5 Financial Support | A5.1 Financial Support and Management | 1. Clear financial records and standardised management systems for palliative care services 2. Establish management systems for personal and social donations |
| B Process Indicators | B1 Palliative Care Admission Assessment Service | B1.1 Admission Criteria | 1. Diagnosed with advanced/terminal-stage cancer or other terminal chronic diseases, with a projected survival period ≤6 months, or in a decompensated phase of illness; individuals with a demonstrated need for services who voluntarily agree to the palliative care service contract |
|  |  | B1.2 Basic Data Collection and Comprehensive Assessment | 1. Basic Data Collection: Includes personal information, family/social relationships and financial status, medical history, and recent physical examination reports to establish health records for older residents 2. Comprehensive Assessment: Includes evaluation of cognitive function, functional status, nutritional screening, symptoms, pain, psychological condition, and social support to determine care level |
|  | B2 Comfort Care Services | B2.1 Basic Nursing Services | 1. Regularly assess and develop personalised basic care services, providing a comfortable environment, assistance with bathing, oral care, feeding support, enteral and parenteral nutrition care, skin care, positioning care, incontinence management, perineal care, and maintaining timely documentation |
|  |  | B2.2 Catheter Care | 1. Regularly assess catheter patency, fixation, and surrounding skin condition. Provide services such as indwelling urinary catheter management, with timely documentation |
|  | B3 Symptom Management Services | B3.1 Symptom Assessment and Management | 1. Conduct regular dynamic assessments of older residents' symptoms, providing management services for pain, dyspnoea, coughing, expectoration, haemoptysis, oedema, fever, delirium, etc., with timely documentation 2. Select appropriate pharmacological and non-pharmacological interventions based on the symptoms of older residents, paying attention to medication timing, dosage, administration methods, efficacy monitoring, and observation of adverse reactions |
|  | B4 Death Education Services | B4.1 Death Education Content | 1. Deliver personalised death education tailored to the specific needs of the elderly person and their family, covering fundamental knowledge about death, attitudes towards death and dying, exploring life's meaning, the Four Stages of Life, advance directives, and other relevant educational content |
|  |  | B4.2 Methods and Approaches to Death Education | 1. Select targeted educational methods and channels based on the actual circumstances of the elderly person and their family, including face-to-face discussions, provision of palliative care written materials and information booklets, and hosting family meetings |
|  | B5 Psychological Support and Humanistic Care | B5.1 Communication with Older Residents and Their Families | 1. Healthcare personnel shall inform the elderly person or their family of disease progression using appropriate methods, facilitating shared decision-making communication while safeguarding family privacy and documenting implementation details 2. Palliative care service personnel shall establish effective communication mechanisms with older residents and their families, assisting the elderly in formulating advance medical directives or living wills to accommodate their treatment preferences 3. Encourage end-of-life discussions between the elderly person and their family, documenting the implementation process 4. Communication shall respect cultural diversity |
|  |  | B5.2 Psychological Support and Humanistic Care for Older Residents and Their Families | 1. Regularly assess and address negative psychological states and emotions (such as anxiety, depression, grief) among older residents and their families, assisting them in adjusting to and coping with issues surrounding mortality, with timely documentation 2. Assess spiritual needs of older residents and their families, guiding the elderly to formulate realistic wishes and complete unfinished matters. Address these according to their religious beliefs and ethnic customs, documenting implementation 3. Facilitate family relations, encouraging relatives to provide loving companionship to the elderly and alleviate physical and emotional distress |
|  |  | B5.3 Protection of Rights | 1. Safeguard the elderly person's legal rights, including the right to life, right to informed consent, right to privacy, and right to self-determination 2. Ensure equitable treatment for the elderly throughout palliative care services |
|  |  | B5.4 Social Support Services | 1. Seek social support such as welfare assistance and policy support based on the circumstances of the elderly person and their family 2. Safeguarding older residents' rights to healthcare coverage and social welfare benefits, alongside coordination of social resources |
|  | B6 Post-Death Arrangements and Bereavement Care Services | B6.1 Post-Death Arrangement Services | 1. Assisting families in preparing matters following the elderly person's passing (e.g., funeral attire, funeral arrangements) 2. Conduct funeral arrangements in accordance with the wishes and customs of the elderly person and their family 3. Death registration, body care (including cosmetic care, terminal disinfection of the residence, and identification), body transportation, and encouraging family participation in body care |
|  |  | B6.2 Bereavement Care Services | 1. Implementation of comprehensive bereavement care and grief counselling programmes (including bereavement risk assessment, individual counselling, online support, etc.) |
|  | B7 Palliative Care Transfer Services | B7.1 Transfer Services and Management | 1. Includes verification of handover details, notification of transfer, settlement of fees, and archiving of records 2. Conduct monitoring and management of symptoms and signs following patient discharge, develop follow-up plans, and provide palliative care guidance |
| C Outcome Indicators | C1 Comfort Care Outcomes | C1.1 Quality of Basic Nursing | 1. Implementation rate of comfort environment management (%) = Number of fully correct (or complete) items checked / (Total number of items checked – Number of items not applicable) × 100% 2. Implementation rate of basic care = Number of fully implemented items / Total number of items required × 100% 3. Basic Care Compliance Rate = Number of palliative care older residents meeting standards / total number of palliative care older residents inspected × 100% 4. Hygiene Care Quality Compliance Rate = Number of palliative care older residents meeting hygiene care quality standards / Total number of palliative care older residents inspected × 100% 5. Excretion Care Quality Compliance Rate = Number of palliative care older residents with compliant excretion care quality / Total number of palliative care older residents inspected × 100% |
|  |  | C1.2 Quality of Catheter Care | 1. Catheter Risk Assessment Rate = Number of catheterised palliative care older residents who completed risk assessment / Total number of catheterised palliative care older residents sampled × 100% 2. Catheter Management Implementation Rate (%) = Number of fully correct (or complete) items checked / (Total number of items checked – Number of items not applicable) × 100% |
|  | C2 Quality of Symptom Management | C2.1 Quality of Symptom Assessment and Management | 1. C-PPS is recommended for assessing physical functional status in palliative care older residents 2. C-NRS is recommended for assessing pain relief in palliative care older residents 3. C-ESAS is recommended for assessing symptoms such as fatigue, nausea, appetite, dyspnoea, and sleep disturbance in elderly palliative care older residents 4. NRS-2002 is recommended for assessing nutritional status in elderly palliative care older residents |
|  | C3 Quality of Death Education | C3.1 Quality of Death Education for Older Residents | 1. The C-PCAS-9 is recommended for assessing elderly palliative care older residents' attitudes towards palliative care 2. The C-MLQ is recommended for assessing elderly palliative care older residents' perception of life meaning 3. The C-DAP-R is recommended for assessing elderly palliative care older residents' attitudes towards death |
|  |  | C3.2 Quality of Death Education for Family Members | 1. The C-PaCKS is recommended for assessing family members' level of understanding regarding palliative care 2. The C-DAP-R is recommended for assessing the death attitudes of family members of elderly palliative care older residents |
|  | C4 Quality of Psychological Support and Humanistic Care | C4.1 Quality of Communication with Older Residents and Their Families | 1. Shared decision-making rate = Number of families making shared decisions / Number of families holding family meetings × 100% 2. Palliative care document signing rate = Number of older residents signing palliative care documents / Total number of older residents receiving palliative care × 100% |
|  |  | C4.2 Quality of Psychological Support and Humanistic Care for Older Residents and Their Families | 1. The DASS-21 and the DADDS-C are recommended for measuring psychological distress relief among older residents and families 2. The fulfillment of advance directives and final wishes for residents receiving palliative care 3. The presence of family members at the bedside during the older resident's final moments |
|  |  | C4.3 Status of Rights Protection and Social Support | 1. Respect and fulfilment of reasonable needs of palliative care older residents and their families 2. Availability of social resource support (e.g., social workers, volunteers) for palliative care older residents and their families |
|  | C5 Outcomes of Post-Death Arrangements and Bereavement Care | C5.1 Quality of Post-Death Arrangements | 1. Compliance with standards for the preparation of the deceased 2. Assistance in the smooth completion of funeral arrangements |
|  |  | C5.2 Quality of Bereavement Care Services | 1. Assistance in managing bereavement-related matters 2. The C-BRAT is recommended for measuring bereavement risk levels |
|  | C6 Palliative Care Transfer Service Outcomes | C6.1 Quality of Transfer Services and Management | 1. Clear and streamlined transfer service procedures 2. Implementation of a follow-up management system, with quality assessment based on follow-up content |
|  | C7 Incidence of Adverse Events | C7.1 Incidence of Falls/Bed Falls, Pressure Injuries, Unplanned Extubation and Other Adverse Events | 1. Incidence rate of falls/bed falls among palliative care residents = Number of falls or bed falls among older residents receiving palliative care during the same period / Actual bed days occupied by older residents receiving palliative care during the statistical period × 1000‰ 2. Incidence rate of stage 2 or higher pressure injuries among palliative care older residents = Number of new in-hospital pressure injuries of stage 2 or higher among older residents during the same period / (Total number of palliative care older residents at the start of the statistical period + Total number of new palliative care older residents during the period) × 100% 3. Rate of unplanned catheter removal in palliative care older residents with indwelling catheters = Number of unplanned catheter removals during the period / Total days of catheter indwelling within the statistical period × 1000‰ 4. Incidence rate of other adverse events in palliative care older residents = Number of other adverse events (e.g., burns, choking, aspiration, suicide attempts, medication errors) during a period / Actual bed-days occupied by palliative care older residents during the statistical period × 100% |
|  | C8 Infection Control Quality | C8.1 Healthcare-Associated Infection Status | 1. Incidence rate of healthcare-associated infections = Number of palliative care older residents with new healthcare-associated infections / Total number of palliative care older residents in the institution during the same period × 100% 2. Catheter-associated urinary tract infection incidence rate (‰) = Number of catheter-associated urinary tract infections occurring during the same period / Total catheter-in-place days within the statistical period × 1000‰ |
|  |  | C8.2 Implementation of Healthcare-Associated Infection Prevention Measures | 1. Sterile Technique Compliance Rate (%) = Number of compliant sterile technique procedures / Total number of sterile technique procedures sampled × 100% 2. Hand hygiene compliance rate (%) = Number of hand hygiene interventions performed / Number of required hand hygiene interventions × 100% 3. Implementation Rate of Catheter-Associated Urinary Tract Infection Prevention Management = Number of fully correct (or complete) inspection items / (Total number of inspection items - Number of inapplicable items) × 100% |
|  |  | C8.3 Communicable Disease Prevention Status | 1. Prevention systems and contingency plans for infectious diseases and other public health events are in place |
|  | C9 Documentation Management | C9.1 Quality of Medical Care Documentation | 1. Compliance rate for medical documentation (e.g., end-of-life decision-making communications, medical condition records, resuscitation records, assessment forms, etc.) = Number of compliant medical records / Total number of medical records reviewed × 100% |
|  | C10 Feedback and Quality Improvement | C10.1 Feedback from Older Residents and Their Families | 1. Service satisfaction among older residents and their families 2. Number of complaints from older residents and their families |
|  |  | C10.2 Staff Feedback | 1. Staff satisfaction with the organisation 2. Self-assessment by service staff 3. Staff suggestions and opinions regarding work |
|  |  | C10.3 Institutional Quality Improvement | 1. Institution complaint management includes self-assessment, review, improvement and documentation 2. Complaint management implementation rate (%) = Number of fully implemented items / (Total number of items checked – Number of items not applicable) × 100% 3. Quality management organisation established to conduct service quality evaluations |

Note: C-PPS, Chinese Version of Palliative Performance Scale; C-NRS, Chinese Version of Numerical Rating Scale; C-ESAS, Chinese version of the Edmonton Symptom Assessment System (Revised); NRS-2002, Nutrition Risk Screening 2002; C-PCAS-9, Chinese Version of Palliative Care Attitudes Scale; C-MLQ, Chinese Meaning in Life Questionnaire; C-DAP-R, Chinese Version of the Death Attitude Profile-Revised; C-PaCKS, Chinese Version of the Palliative Care Knowledge Scale; DASS-21, Depression, Anxiety and Stress Scale (Chinese Version); DADDS-C, Chinese Version of the Death and Dying Distress Scale; C-BRAT, Chinese Version of the Bereavement Risk Assessment Tool.
